# Distribution, Sources, and Risks of Heavy Metal Contamination in Farmland Soils Surrounding Typical Industrial Areas of South Shanxi Province, China

**DOI:** 10.3390/toxics13110984

**Published:** 2025-11-16

**Authors:** Ying Zhao, Yirong Ren, Fei Wang

**Affiliations:** 1 Department of Chemistry and Chemical Engineering, Jinzhong University, Jinzhong 030619, China; shadowying210@163.com (Y.Z.); renyirong621@163.com (Y.R.); 2 School of Life Science, Shanxi University, Taiyuan 030006, China

**Keywords:** soil, heavy metals, source apportionment, risk assessment

## Abstract

This research investigated the characteristics and risks associated with heavy metal contamination in farmland soils surrounding an industrial aggregation area in Yuncheng City, southern Shanxi Province. It analyzed the concentrations and spatial accumulation patterns of eight heavy metal elements, employed principal component analysis (PCA) to identify sources, and assessed both the ecological and health risks. The results revealed the following: (1) The mean concentrations of Pb, Cd, As, Hg, Cr, Zn, Cu, and Ni in the study area were 26.1, 0.29, 13.4, 0.05, 61.4, 72.94, 27.15, and 32.33 mg·kg^−1^, respectively. These concentrations were elevated above local background levels but remained within Chinese regulatory thresholds for agricultural soil. According to the geoaccumulation index, only Cd was classified as slightly polluted, while the other elements were essentially uncontaminated. The Nemerow comprehensive pollution index indicated light pollution. The potential ecological risk index identified Cd and Hg as the primary contributors to ecological risk, indicating moderate contamination. (2) Source apportionment results revealed that As, Cr, and Ni originated from industrial–natural sources; Cd, Zn, and Cu were linked to industrial production; and Pb and Hg were attributed to mining and traffic sources. (3) Health risk assessments suggested that non-carcinogenic risks for adults and children (0.28 and 0.51, respectively) were within safe limits. However, the carcinogenic risk for children (1.02 × 10^−4^) exceeded the acceptable threshold, while the level for adults (4.67 × 10^−5^) remained acceptable. This study provides a scientific basis for preventing, controlling and remediating local heavy metal contamination in soil.

## 1. Introduction

Against the backdrop of accelerating global industrialization and urbanization, the severity of soil heavy metal contamination is highlighted by its substantial risks to ecological stability and public health [1]. Pb, Hg, Cd, Cr, and As are known for their persistence and bioaccumulation. These metals remain in soil for extended periods, becoming enriched through the food chain and endangering ecosystems and human health [2,3]. The primary sources of these heavy metals include industrial discharges, improper agricultural practices (such as the use of pesticides and fertilizers), and sewage irrigation [4,5]. Once soil becomes contaminated with heavy metals, its fertility declines, and the composition and function of microbial communities within the soil are disrupted, upsetting the balance of the soil ecosystem [6,7]. For humans, chronic exposure to heavy metals through contaminated agricultural products poses significant health risks, including damage to the digestive, nervous, and cardiovascular systems, and an increased risk of cancer [8].

Currently, considerable research focuses on the risks of heavy metals in soils surrounding industrial enterprises, primarily addressing pollution characteristics, risk levels, and source apportionment. A study of surface soils around a coal-fired power station in India revealed that the concentrations of Fe and Ni exceeded WHO and FAO standards both before and after the monsoon season. The presence of As and Cd posed moderate to relatively high ecological risks [9]. Research in Yasi County, Romania, indicated that heavy metals in the local soil threatened both human health and the ecosystem, with Cr exceeding standards in 45.45% of samples, while Hg, As, and Pb exceeded limits in some protected areas [10]. A study of the topsoil in the Upper Ohře River Basin (Czech Republic and Germany) showed considerable variability in concentrations of As, Cd, Cu, Pb, and Zn across the area, with levels exceeding the legislative limits of the Czech Republic. The potential ecological risk index indicated a strong risk, while the health assessment found no significant health risks (non-carcinogenic and carcinogenic) for adults. In contrast, children were identified as being at risk, primarily due to As [11]. A study of surface wetland soils in the Yellow River Delta found elevated levels of Hg and As, with potential ecological risks primarily at moderate to strong levels. Source apportionment identified three main contributors: natural sources (32%), industrial sources (45%), and agricultural sources (23%). Human health risk analysis indicated that carcinogenic risks for both adults and children were mainly attributed to As and Ni, with the carcinogenic risk index for As exceeding the safety threshold of 10^−6^ in some areas [12]. An investigation of a red mud disposal site in western Shanxi Province revealed that soil concentrations of Al, Fe, Na, Cr, and Pb were significantly higher than background levels, with Cr exhibiting the highest contamination factor. Both Cr and Cd demonstrated high potential ecological risk coefficients, and their long-term accumulation could threaten the safety of surrounding crops [13]. In a typical mining and smelting industrial zone in southwestern China, levels of Cd, Cu, Zn, and Pb in the nearby agricultural soils exceeded the risk limits for agricultural soil pollution established in China. This pollution was primarily attributed to a combination of industrial and agricultural activities. Health risk assessments indicated that, while non-carcinogenic risks for adults and children were low, attention should be directed toward the cancer incidence posed by Cd, Pb, and particularly Ni [14]. Soils in a typical suburban industrial area of western Laizhou, Shandong, exhibited significant heavy metal pollution and environmental risk, largely due to industrial production activities and other anthropogenic factors contributing to heavy metal enrichment [15]. Near a coal-fired power plant in western China, certain toxic metal elements posed potential risks, likely due to emissions from the coal-fired power generation process [16]. In summary, while these surveys have focused on heavy metal pollution in soils surrounding various industrial areas, there is still a lack of systematic research on farmland soils near enterprises involved in metal mining, smelting, processing, and diversified industrial agglomeration.

Yuncheng City, a key location in the “Ecological Protection and High-Quality Development Plan for the Yellow River Basin,” has both industrial characteristics and ecological importance. The surrounding areas often host various industries, including metal mining and smelting. Consequently, concentrations of heavy metals often exceed regional background values, with some areas exhibiting pollution that poses risks to the soil ecosystem and agricultural product safety. This research aims to systematically analyze heavy metal pollution in the farmland soils surrounding representative enterprises, accurately identify the sources of this pollution, and assess the associated ecological and health risks. The findings are crucial for preventing heavy metal contamination in farmland soils near industrial and mining areas, ensuring the safe use of cultivated land, and guaranteeing the safety and quality of agricultural products in Shanxi Province.

## 2. Materials and Methods

### 2.1. Study Area

The research area is situated in Yuncheng City, in the southern part of Shanxi Province. It features a warm temperate monsoon climate, with an average annual temperature of 13.5 °C and precipitation 550 mm. The area benefits from convenient transportation and a strong industrial base, establishing it as a key hub for the metal smelting and processing industry in Shanxi Province. Numerous enterprises operate across various sectors, including mining, iron and steel smelting, magnesium and magnesium alloy production, metal processing, and metallurgical auxiliary materials. This has led to in a relatively complete industrial chain that encompasses mineral resource development, smelting processing, and end-product manufacturing, forming a core pillar of the regional economy. The primary agricultural land in the area is dry cropland. The predominant soil type in this region is cinnamon soil, classified as Cambisols in the International World Reference Base for Soil Resources (WRB) 2022 system [17,18]. The types of soil parent materials in study area are mainly aeolian parent materials (loess), which are characterized by uniform texture. The primary soil texture is loam soil, with the sand, silt, and clay fractions being 41%, 44% and 15%, respectively. The average pH, organic matter and TN contents are 8.1, 17.18 g/kg and 1.12 g/kg, respectively [19]. Due to the uniform texture of soil, the physical and chemical properties presented almost no difference in different sampling sites. Overall, the research area is located in a flat terrain with thick plow layers, moderate texture, good permeability and tillage properties, easy mineralization of organic matter and wide suitability for planting.

### 2.2. Sampling and Analytical Methods

**Figure 1:**
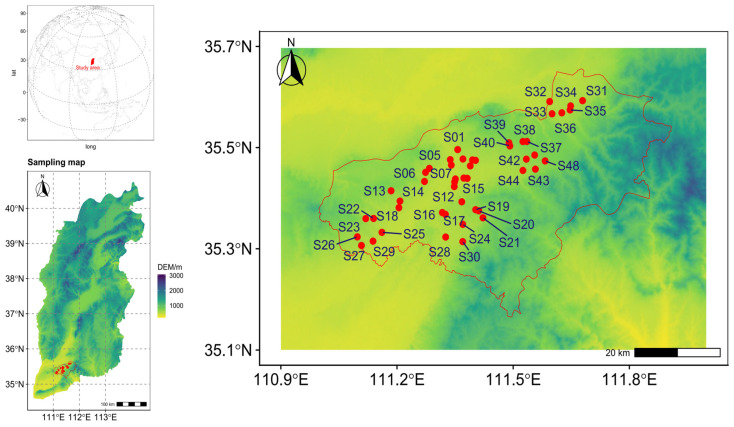
Geographic layout of the sampling points.

Forty-eight soil samples were collected from the agricultural lands adjacent to the enterprise, taking into account the production layout, terrain characteristics, and prevailing wind direction in the factory area (Figure 1). A plastic spade was used to gather topsoil from a depth of 0–20 cm in September 2024. The types of soil parent materials in the study area are mainly aeolian parent materials (loess), which are characterized by uniform texture. The soil formed by the weathering process of parent material has a more significant accumulation of heavy metals (such as Cd and Pb) in the surface layer. In addition, the tillage layer is highly representative, serving as the main distribution area for crop roots and the active zone where most biogeochemical processes occur. Furthermore, the “Soil Environmental Quality Standards” and “Agricultural Land Risk Control Standards” in China and many other countries specify a sampling depth of 0–20 cm in the cultivated layer. This guideline directs the majority of environmental monitoring and investigation projects to concentrate resources and attention on this depth to meet regulatory requirements and facilitate horizontal comparisons.

After collection, samples were immediately sealed in labeled PVC packages for transport. The subsequent procedures involved air-drying the samples, removing gravel or plant roots, and passing them through a 2 mm sieve to remove plant debris and other impurities. Then, these samples were ground in a porcelain mortar and sieved through a 100-mesh screen for laboratory analysis.

To measure Cd and Pb contents, a sample weighing 0.2–0.5 g (accurate to 0.1 mg) was heated and subjected to acid digestion using a mixture of HNO_3_, HCl, HF and HClO_4_ until the solution evaporated to a viscous state. The solution was then cooled and brought to the marked volume for testing. Detection was performed using graphite furnace atomic absorption spectrophotometry (GFAAS) with an Agilent 240Z AA atomic absorption spectrometer (Agilent Technologies, Santa Clara, CA, USA) [20]. The instrument detection limits for Cd and Pb were 0.01 mg/kg and 0.1 mg/kg, respectively. For the determination of Hg and As, samples weighing 0.2–1.0 g with 0.1 mg accuracy) underwent acid digestion using a HNO_3_-HCl mixture. After cooling, the digestates were diluted to the appropriate volume and analyzed using a Jitian AFS-8530 atomic fluorescence spectrometer (Beijing Jitian Instrument Co., Ltd., Beijing, China) [21,22]. The method achieved detection limits of 0.01 mg/kg for As and 0.002 mg/kg for Hg. The pre-treatment method for Zn, Cr, Cu, and Ni involved heating and digesting a 0.2–0.3 g sample (accurate to 0.1 mg) with a mixture of HNO_3_, HCl, HF and HClO_4_. Their contents were analyzed using flame atomic absorption spectrophotometry (FAAS) with an Agilent 240FS AA atomic absorption spectrometer (Agilent Technologies, USA) [23]. The detection limits for these metals were 1 mg/kg for Zn, 4 mg/kg for Cr, 1 mg/kg for Cu, and 3 mg/kg for Ni.

Quality control measures were strictly implemented in accordance with the “Chinese Technical Specification for Soil Environmental Monitoring” (HJ/T 166-2004) [24]. During field sampling, duplicate samples were collected from 10% of the samples in each batch. For laboratory analysis, duplicate samples and certified reference materials were tested for 20% of the samples in each batch. Additionally, laboratory blank samples were analyzed simultaneously as part of our quality assurance protocol.

### 2.3. Risk Assessment of Heavy Metal Indices

#### 2.3.1. The Geoaccumulation Index

The geo-accumulation index is a widely utilized used for assessing soil heavy metal [25]. It distinguishes itself by considering both the natural geochemical background and contributions from anthropogenic pollution. The formula for its calculation is

(1) $$I_{\mathrm{geo}}={\;\log}_{2}(\frac{C_{n}}{{1.5B}_{n}})$$

where *C_n_* represents the heavy metal content (mg/kg) and *B_n_* denotes the geochemical background value (mg/kg), as presented in Supplementary Table S1 [26]. These values are from the publication of China National Environmental Monitoring Centre. In Shanxi Province, 255 soil samples were collected and measured. So, the background value is an average value of these soil samples. The constant 1.5 is introduced to account for variations in the background value. The classification criteria for Equation (1) are provided in Supplementary Table S2.

#### 2.3.2. The Nemerow Comprehensive Index

The Nemerow index serves as a comprehensive measure of soil environmental quality by integrating the combined effects of multiple heavy metals, with particular sensitivity to peak concentrations that disproportionately influence the overall assessment [27,28]. It can be expressed as follows:

(2) $$P_{i}\;=\;\frac{C_{i}}{S_{i}}$$

(3) $$P_{N}=\sqrt{\frac{P_{\mathrm{imax}\;}^{2}+P_{\mathrm{avg}\;}^{2}}{2}}$$

where *P_i_* represents the individual heavy metal pollution index, *C_i_* denotes the measured content of the heavy metal in the soil (mg·kg^−1^); *S_i_* refers to the background value of the soil element (mg·kg^−1^); *P_max_* indicates the maximum value; *P_avg_* signifies the mean of the indices for the soil samples, and *P_N_* is the Nemerow comprehensive index. The pollution classification criteria are detailed in Table S2 [29,30].

#### 2.3.3. The Potential Ecological Risk Index

The potential ecological risk index is a comprehensive methodology introduced by Swedish scholar Hakanson. It evaluates risks by integrating heavy metal concentrations with their respective ecological, environmental, and toxicological impacts [31]. The equations are as follows:

(4) $$E_{r}^{i}=T_{r}^{i}\;\times \;\frac{C_{i}}{S_{i}}$$

(5) $$\mathrm{RI}=\sum_{i=l}^{n}E_{r}^{i}$$

where $E_{r}^{i}$ is the potential ecological risk index for an individual heavy metal, and the toxic response factors are 40 for Hg, 30 for Cd, 10 for As, 5 for Pb, 5 for Ni, 5 for Cu, 2 for Cr, and 1 for Zn. *C_i_* represents the measured heavy metal contents (mg/kg), while *S_i_* denotes the background value (mg/kg). *RI* is the integrated potential ecological risk index. The classification criteria are presented in Table S2 [32,33].

#### 2.3.4. Health Risk Assessment

The Hazard Index (HI) and Carcinogenic Risk (CR), following U.S. Environmental Protection Agency guidelines, were used to evaluate both non-carcinogenic and carcinogenic human health risks [34]. The primary exposure pathways for heavy metals in soil include ingestion, dermal absorption, and inhalation [35], calculated as follows:

(6) $${\mathrm{ADD}}_{\mathrm{ing-soil}}\;=\;\frac{C_{s}\;\times \;{\mathrm{IngR}}_{s}\;\times \;\mathrm{EF}\;\times \mathrm{ED}}{\mathrm{BW}\times \mathrm{AT}}\times 10^{-6}$$

(7) $${\mathrm{ADD}}_{\mathrm{der-soil}}=\frac{C_{s}\;\times \;\mathrm{SA}\;\times \mathrm{AF}\;\times \;\mathrm{ABS}\;\times \mathrm{EF}\;\times \;\mathrm{ED}}{\mathrm{BW}\;\times \;\mathrm{AT}}\times 10^{-6}$$

(8) $${\mathrm{ADD}}_{\mathrm{in}h\mathrm{-soil}}=\frac{C_{s}\;\times \;{\mathrm{In}hR}_{s}\;\times \;\mathrm{EF}\;\times \;\mathrm{ED}}{\mathrm{PEF}\;\times \;\mathrm{BW}\;\times \;\mathrm{AT}}$$

where ${\mathrm{ADD}}_{\mathrm{ing-soil}}$, ${\mathrm{ADD}}_{\mathrm{der-soil}}$, ${\mathrm{ADD}}_{\mathrm{in}h\mathrm{-soil}}$ represent the Average Daily Dose from soil ingestion, dermal contact with soil, and inhalation of soil particles, respectively (mg/kg/day); *C_s_* denotes the concentration of the heavy metal in the soil (mg/kg). The values for other exposure parameters are listed in Supplementary Table S3 [36,37].

The non-carcinogenic risk can be calculated as

(9) $$\mathrm{HI}=\sum {\mathrm{HQ}}_{i}=\sum \frac{{\mathrm{ADD}}_{\mathrm{ij}}}{{\mathrm{RfD}}_{\mathrm{ij}}}$$

The carcinogenic risk can be calculated as

(10) $$\mathrm{TCR}=\sum {\mathrm{CR}}_{\mathrm{ij}}=\sum {\mathrm{ADD}}_{\mathrm{ij}}\;\times \;{\mathrm{SF}}_{\mathrm{ij}}$$

where *i* corresponds to the number of individual metal elements, and *j* refers to their exposure pathways. The reference dose (*RfD*, mg/kg/day) and slope factor (*SF*, mg/kg/day) indicate the toxicity thresholds for each metal via specific exposure routes. The hazard quotient (*HQ*) quantifies the non-carcinogenic risk of a single element, while the hazard index (*HI*) aggregates the *HQ* values of all elements to represent the overall non-carcinogenic risk. An *HI* value below 1 implies negligible risk, while a value exceeding 1 suggests potential adverse effects. Similarly, *CR* reflects the carcinogenic risk of a single metal across all pathways, and *TCR* sums the *CR* values of all metals. A *TCR* between 1 × 10^−6^ and 1 × 10^−4^ is considered acceptable; values above 1 × 10^−4^ indicate a significant carcinogenic threat, while those below 1 × 10^−6^ denote minimal risk. Specific RfD and SF values used in this study are provided in Table S4 [36,37].

### 2.4. Statistical Method

All statistical procedures and graphical representations were conducted in R (version 4.5.0, available at https://cran.r-project.org, accessed on 1 September 2025). Data preprocessing and integration were managed using the “tidyverse” suite of packages, while descriptive summaries were generated with the “gtsummary” package. Correlation analyses were performed using the “ggcor” package. Spatial interpolation based on the inverse distance weighting (IDW) method was carried out with the “sf” and “rspatial” packages. All visualizations were created using with the “ggplot2” library within the R computational framework.

## 3. Results

### 3.1. Description and Spatial Distribution of Heavy Metals in Study Area

Table 1 presents the concentrations of Pb, Cd, As, Hg, Cr, Zn, Cu, and Ni in soil, with values ranging from 20.10 to 34.80, 0.14 to 2.02, 10.80 to 16.00, 0.010 to 0.190, 51.00 to 81.00, 59.02 to 120.10, 19.03 to 49.22, and 23.10 to 44.30 mg·kg^−1^, respectively. The results reveal distinct variations among the elements: Cd and Hg exhibit extremely high spatial variability, indicating significant influence from anthropogenic point source pollution, which leads to uneven spatial distribution. This underscores the pronounced impact of human activities on specific heavy metal contamination [38]. Hg, As, and Pb show slight to moderate enrichment with differentiated spatial dispersion. Hg is particularly affected by localized industrial emissions, while As and Pb are influenced by diffuse sources such as agricultural activities and atmospheric deposition, resulting in a relatively uniform pollution distribution. Cu, driven by dispersed diffuse sources, exhibits moderate enrichment and dispersion. In contrast, Zn, Ni, and Cr are primarily governed by natural background levels, with minimal anthropogenic influence. In summary, although the average levels of these elements exceed the background values for soil heavy metals in Shanxi Province, they remain below the Chinese risk threshold values for soil contamination of farmland [39]. Soil pH, redox potential directly or organic matter content affect the availability and behavior of heavy metals [40,41,42,43]. The soil pH in study area is 8.1, which belongs to light-alkalinity. The surface of soil colloids becomes more negatively charged, enhancing its capacity to adsorb metals. The soil texture is mainly loamy soil. The soil type is dryland, with good ventilation and in an oxidized state, which may decrease the solubility and mobility of most heavy metals. The content of soil organic matter is 17.18 g/kg, which is a good level. It may immobilize heavy metals through adsorption or the formation of stable organic complexes, thereby reducing metal mobility in the soil.

**Figure 2:**
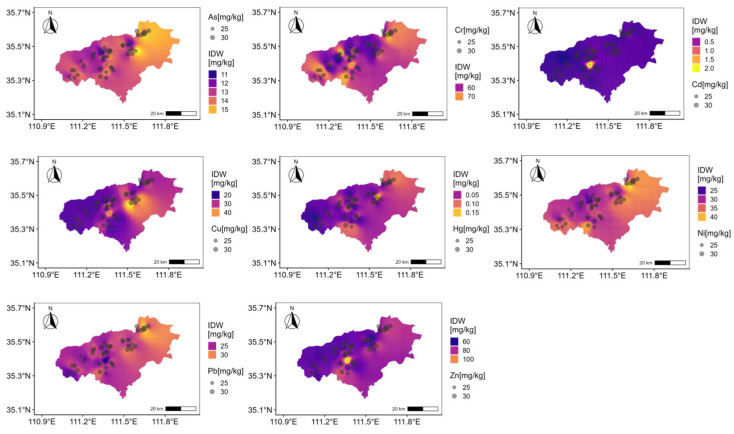
Spatial distribution of heavy metals.

Their spatial distribution reveals distinct regional differentiation (Figure 2). As, Cu, Hg, Ni, and Pb concentrations are predominantly high in the eastern region, while Cr and Zn are concentrated in the central area. In contrast, high concentrations of Cd are patchy and localized, exhibiting strong spatial heterogeneity. Additionally, Cd shows significant enrichment, whereas Pb, As, and Hg display varying degrees of enrichment. Cr levels are slightly below the soil background value.

### 3.2. Source Analysis

As shown in Table 2, the Pearson correlation coefficients for the eight heavy metals revealed a highly significant positive correlation among Pb, As, and Hg (*p* < 0.01). This association is closely related to their co-deposition in the soil through atmospheric sedimentation resulting from Pb fuel combustion in transportation, as well as the emissions of As and Hg pollutants from coal-fired power plants and industrial activities. The convergence of multiple pollution sources leads to the simultaneous accumulation of these three elements in the soil. Additionally, highly significant positive correlations were observed among As, Cr, Zn, Cu, and Ni (*p* < 0.01), indicating a pronounced synergistic accumulation among these elements. This pattern aligns with the simultaneous release of multiple heavy metals from industrial activities and their subsequent enrichment in the soil, as documented in previous studies [44]. The strong correlations confirm their origin from common industrial pollution sources, such as metal smelting and machinery manufacturing, which result in the concurrent input of multiple heavy metals into the soil environment, leading to synergistic accumulation [45]. Furthermore, a significant positive correlation was found between Pb and Ni, as well as between As and Cu (*p* < 0.05). These correlations may arise from the combined influence of traffic and industrial activities, while the relationship between As and Cu is likely attributable to the use of arsenic- and copper-containing mineral fertilizers in agricultural practices, along with the geochemical migration of these elements within the soil.

PCA was further utilized to analyze the sources of the elements. The results in Table 3 indicate that three principal components were extracted, accounting for a cumulative variance contribution rate of 73.84%.

The first principal component (PC1), which accounts for 27.75% of the variance, showed high factor loadings for As, Cr, and Ni. The significant positive correlations among these three heavy metals suggest a common source [46]. Metallurgical and chemical enterprises release elements such as As and Cr through exhaust gas emissions, wastewater, and waste residues generated during production. Additionally, the use of phosphate fertilizers in agriculture can introduce related components into the soil. Consequently, PC1 is identified as an industrial-natural source. The second principal component (PC2), contributing 26.20% to the variance, exhibited prominent factor loadings for Cd, Zn, and Cu. Continuous accumulation of these elements in the surrounding farmland soil is primarily due to wastewater discharge from electroplating plants and waste residues from steel manufacturing. Correlation analysis revealed significant positive correlations among these elements, indicating similar source pathways. Thus, PC2 is identified as an industrial production source. The third principal component (PC3), accounting for 19.89% of the variance, primarily loaded on Pb and Hg. The presence of small mines near the study area significantly contributes to Hg input into the soil through processes such as the crushing and screening of Hg-containing ores during mining. Additionally, the relatively high traffic volume in the area leads to the gradual accumulation of Pb in farmland soil, resulting from Pb dust emitted by vehicle exhaust and dust diffusion from nearby metal processing enterprises. The low loadings of Pb and Hg in PC1 and PC2 suggest relatively independent and specific sources. Therefore, PC3 is identified as a mining-traffic source.

### 3.3. Ecological Risk Assessment

As detailed in Table 4, the geo-accumulation index indicates that the mean contents of Pb, As, Hg, Cr, Zn, Cu, and Ni are less than 0, signifying an uncontaminated state. However, some sampling points exhibit contamination. The mean value of Cd is 0.64, indicating slight contamination, which may be attributed to the discharge of Cd-containing waste from industrial production activities. Additionally, Cd has high mobility in soil and tends to accumulate. Furthermore, Hg readily mixes with organic matter in soil to form stable compounds [47]. Although Pb and As show no overall contamination, some sampling points indicate slight contamination, possibly due to atmospheric deposition from industrial exhaust emissions or the use of wastewater containing these heavy metals for agricultural irrigation [48,49]. The results of the single factor index reveal that Cd is the primary contributor to contamination, classified as slightly contaminated, while other metals remain uncontaminated. Similarly, the single potential ecological risk index results demonstrate that Cd and Hg pose moderate risks, while other metals are classified as low-risk. This is directly related to the discharge of Cd and Hg-containing wastewater and exhaust from nearby chemical plants, as well as Hg release during certain alloy processing activities [50,51]. Moreover, Cd’s high bioavailability poses a relatively significant risk to the environment.

**Figure 3:**
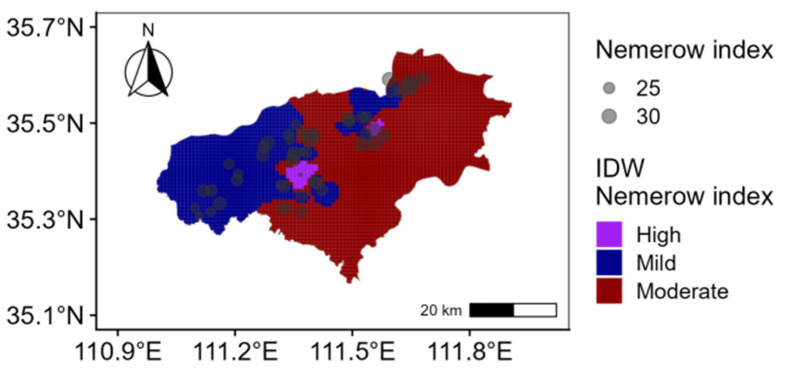
Spatial distribution of Nemerow index.

**Figure 4:**
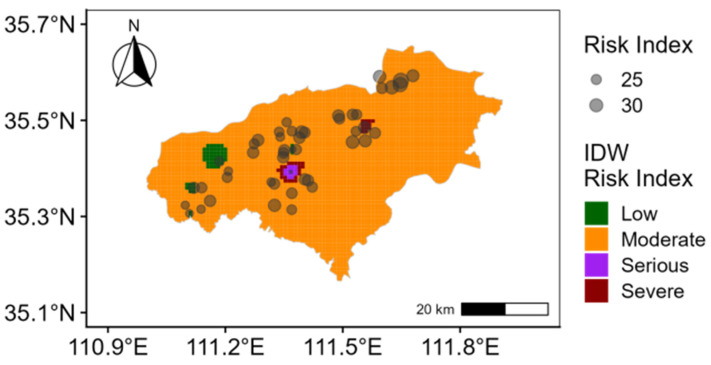
Spatial distribution of potential ecological risk index.

The Nemerow comprehensive pollution index indicates that heavily polluted and moderately polluted points represent 4.2% and 35.4%, respectively, while lightly polluted areas account for 60.4% (Figure 3). The study area is primarily lightly polluted, with localized regions of severe pollution. In terms of the potential ecological risk index, 2.08% sampling points face a severe risk, 2.08% a high risk, 87.50% a moderate risk, and 8.33% a low risk (Figure 4). Overall, the predominant risk level is moderate. This finding is closely associated with the combined effects of highly polluting elements, such as Cd and Hg, particularly in areas concentrated near chemical plants and certain metal processing enterprises [52,53]. Therefore, targeted prevention and control measures should be developed for these high-risk elements and surrounding enterprises to mitigate the comprehensive pollution risk in agricultural soil.

### 3.4. Heath Risk Assessment

The non-carcinogenic risk for heavy metals varies across different exposure pathways. As shown in Table 5, the HI is 0.28 for adults and 0.51 for children, both of which are below 1, indicating a low risk level. Analysis of the various intake pathways reveals that for adults, the combined risk value from all heavy metals through ingestion is 0.074, accounting for 26.55% of the total risk. Dermal contact poses a risk of 0.204, contributing 73.33%, making it the dominant exposure pathway for adults, while inhalation contributes only 0.12%. This indicates that non-carcinogenic risks for adults primarily arise from dermal contact, with inhalation having a negligible impact. In children, the risk through ingestion is 0.173, accounting for 34.02% of the total risk. Dermal contact poses a risk of 0.334, contributing 65.95%, while inhalation contributes only 0.03%. The comparison between adults and children shows similar risk exposures, with dermal contact being the primary pathway for both groups. However, the relative contribution of ingestion is higher in children than in adults, suggesting that children face more significant potential risks from ingestion exposure due to their behavioral patterns [54,55].

The carcinogenic risk associated with the eight heavy metals through various exposure routes is summarized in Table 6. The results indicate that the TCR for adults is 4.67 × 10^−5^, suggesting a potential risk, while for children, it is 1.02 × 10^−4^, exceeding the 1 × 10^−4^ threshold and indicating a significant carcinogenic risk. Analysis of the different intake pathways reveals that ingestion is the dominant route of carcinogenic risk for adults, with a risk index of 3.68 × 10^−5^, accounting for 78.78% of the total risk. Dermal contact contributes a risk value of 9.54 × 10^−6^, representing 20.40% of the total risk. Due to extremely low exposure levels of heavy metals through inhalation, this pathway contributes only about 0.82%. These results highlight that ingestion plays a critical role in carcinogenic threats to adults [56]. For children, the carcinogenic risk via ingestion is 8.59 × 10^−5^, contributing 84.47% of the total risk. Dermal contact presents a value of 1.56 × 10^−5^, accounting for 15.35%, while inhalation has a risk index of 1.78 × 10^−7^, contributing approximately 0.17%. A comparison with adults shows that the distribution of exposure pathways for carcinogenic risk is highly similar between children and adults, with ingestion being the primary source in both groups. However, children face a greater carcinogenic risk than adults and are subject to more potential carcinogenic threats due to their physiological characteristics [57].

## 4. Conclusions

This study evaluated the contamination characteristics, sources, and ecological and health risks of eight heavy metals in agricultural soils surrounding an industrial area. The primary conclusions can be summarized as follows:

Cd was the predominant contaminant in the study area. Although average levels of all heavy metals were below Chinese risk control thresholds for farmland soil, Cd exhibited significant enrichment and strong spatial variability. Hg, As, and Pb showed weak to moderate enrichment. The ecological risk assessment indicated that Cd and Hg are the primary pollutants, presenting slight to moderate ecological risks at some sampling points. Areas with high Cd concentrations exhibited a scattered pattern, while other elements were more concentrated. Source apportionment identified that the heavy metals were from industrial production sources, natural, mining and traffic sources. The health risk assessment showed that non-carcinogenic risk for adults and children was within acceptable limits. Carcinogenic risks for adults were acceptable, but there was a high risk for children. Priority should be given to controlling sources of heavy metal contamination from industrial emissions in soil management for this region.

## Figures and Tables

**Table 1:** Statistics of heavy metal contents.

	Pb	Cd	As	Hg	Cr	Zn	Cu	Ni
Min. (mg/kg)	20.10	0.14	10.80	0.01	51.00	59.02	19.03	23.10
Max. (mg/kg)	34.80	2.02	16.00	0.19	81.00	120.10	49.22	44.30
Mean (mg/kg)	26.11	0.29	13.40	0.05	61.40	72.94	27.15	32.33
Median (mg/kg)	26.05	0.24	13.40	0.04	60.00	70.00	24.50	31.50
Standard deviation (SD)	2.72	0.26	1.12	0.03	7.52	10.29	6.25	3.96
Coefficient of variation (CV,%)	10.40	90.79	8.38	69.97	12.24	14.11	23.02	12.24

**Table 2:** Pearson correlation analysis of eight heavy metals in the research area.

Element	Pb	Cd	As	Hg	Cr	Zn	Cu	Ni
Pb	1							
Cd	−0.198	1						
As	0.446 **	0.107	1					
Hg	0.476 **	−0.013	0.142	1				
Cr	0.182	0.211	0.425 **	0.108	1			
Zn	0.143	0.710 **	0.417 **	0.218	0.447 **	1		
Cu	0.23	0.381 **	0.335 *	0.159	0.109	0.584 **	1	
Ni	0.349 *	0.083	0.623 **	0.132	0.492 **	0.458 **	0.448 **	1

Note: significant statistical level * *p* < 0.05; ** *p* < 0.01.

**Table 3:** Principal components analysis of eight heavy metals.

Element	PC1	PC2	PC3
Pb	0.374	−0.119	0.792
Cd	0.000	0.898	−0.209
As	0.798	0.127	0.237
Hg	−0.052	0.151	0.852
Cr	0.749	0.173	−0.082
Zn	0.381	0.850	0.110
Cu	0.233	0.670	0.298
Ni	0.824	0.186	0.175
Variance contribution/%	27.75	26.20	19.89
Cumulative variance contribution/%	27.75	53.95	73.84

**Table 4:** Ecological risk assessment of individual heavy metal.

Elements	Geo-Accumulation Index (*I_geo_*)		Single Factor Index (*P_i_*)		Single Potential Ecological Risk Index ($E_{r}^{i}$)	
	Mean	Level	Mean	Level	Mean	Level
Pb	−0.21	EU	1.31	US	39.17	LR
Cd	0.64	SC	2.62	SC	78.52	MR
As	−0.17	EU	1.34	US	26.80	LR
Hg	−0.46	EU	1.31	US	52.35	MR
Cr	−0.74	EU	0.90	US	1.81	LR
Zn	−0.29	EU	1.24	US	1.24	LR
Cu	−0.25	EU	1.29	US	6.46	LR
Ni	−0.34	EU	1.20	US	5.99	LR

Note: Essentially uncontaminated refers to “EU”; Slightly contaminated refers to “SC”; Uncontaminated to slightly contaminated refers to “US”; Low risk refers to “LR”; Moderate risk refers to “MR”; Low risk refers to “LR”; Moderate risk refers to “MR”.

**Table 5:** Non-carcinogenic risk assessment of heavy metal.

Metal	Adults				Children			
	Risk_ing_	Risk_inh_	Risk_der_	Total	Risk_ing_	Risk_inh_	Risk_der_	Total
Pb	7.30 × 10^−3^	7.16 × 10^−6^	3.77 × 10^−3^	1.11 × 10^−2^	1.70 × 10^−2^	3.34 × 10^−6^	6.16 × 10^−3^	2.32 × 10^−2^
Cd	2.82 × 10^−4^	4.14 × 10^−6^	1.46 × 10^−2^	1.49 × 10^−2^	6.57 × 10^−4^	1.93 × 10^−6^	2.39 × 10^−2^	2.46 × 10^−2^
As	4.37 × 10^−2^	6.43 × 10^−6^	5.53 × 10^−2^	9.90 × 10^−2^	1.02 × 10^−1^	3.00 × 10^−6^	9.06 × 10^−2^	1.93 × 10^−1^
Hg	1.54 × 10^−4^	7.91 × 10^−8^	1.14 × 10^−3^	1.29 × 10^−3^	3.59 × 10^−4^	3.69 × 10^−8^	1.86 × 10^−3^	2.22 × 10^−3^
Cr	2.00 × 10^−2^	3.09 × 10^−4^	1.25 × 10^−1^	1.45 × 10^−1^	4.67 × 10^−2^	1.44 × 10^−4^	2.04 × 10^−1^	2.51 × 10^−1^
Zn	2.38 × 10^−4^	3.50 × 10^−13^	6.17 × 10^−4^	8.55 × 10^−4^	5.55 × 10^−4^	1.63 × 10^−8^	1.01 × 10^−3^	1.57 × 10^−3^
Cu	6.64 × 10^−4^	9.72 × 10^−8^	1.15 × 10^−3^	1.81 × 10^−3^	1.55 × 10^−3^	4.53 × 10^−8^	1.88 × 10^−3^	3.43 × 10^−3^
Ni	1.58 × 10^−3^	2.26 × 10^−7^	3.04 × 10^−3^	4.62 × 10^−3^	3.69 × 10^−3^	1.05 × 10^−7^	4.98 × 10^−3^	8.67 × 10^−3^
Total	7.40 × 10^−2^	3.27 × 10^−4^	2.04 × 10^−1^	2.79 × 10^−1^	1.73 × 10^−1^	1.53 × 10^−4^	3.34 × 10^−1^	5.07 × 10^−1^
Contribution	26.55%	0.12%	73.33%	100.00%	34.02%	0.03%	65.95%	100.00%

Note: Risk_ing_ indicates the risk from soil ingestion, Risk_inh_ indicates the risk from dermal absorprotion, and Risk_der_ indicates the risk from inhalation of soil particles.

**Table 6:** Carcinogenic risk assessment of heavy metal.

Metal	Adults				Children			
	Risk_ing_	Risk_inh_	Risk_der_	Total	Risk_ing_	Risk_inh_	Risk_der_	Total
Cd	4.93 × 10^−8^	8.95 × 10^−11^	1.00 × 10^−6^	1.05 × 10^−6^	1.15 × 10^−7^	4.18 × 10^−11^	1.64 × 10^−6^	1.76 × 10^−6^
As	6.74 × 10^−6^	9.98 × 10^−9^	8.54 × 10^−6^	1.53 × 10^−5^	1.57 × 10^−5^	4.66 × 10^−9^	1.40 × 10^−5^	2.97 × 10^−5^
Cr	3.00 × 10^−5^	3.71 × 10^−7^	—	3.04 × 10^−5^	7.01 × 10^−5^	1.73 × 10^−7^	—	7.03 × 10^−5^
Total	3.68 × 10^−5^	3.81 × 10^−7^	9.54 × 10^−6^	4.67 × 10^−5^	8.59 × 10^−5^	1.78 × 10^−7^	1.56 × 10^−5^	1.02 × 10^−4^
Contribution	78.78%	0.82%	20.40%	100.00%	84.47%	0.17%	15.35%	100.00%

## Data Availability

The data and R codes that support the findings of this study are available upon request from the corresponding author.

## Supplementary Materials

The following supporting information can be downloaded at https://www.mdpi.com/article/10.3390/toxics13110984/s1, Table S1: The geochemical background value for heavy metals; Table S2: The classification standards for soil heavy metal pollution; Table S3: Parameters and descriptions for the health risk assessment; Table S4: Reference dose (RfD) and slope factor (SF) of heavy metals from different pathways.
