# Supplementary material for: Distribution, Sources, and Risks of Heavy Metal Contamination in Farmland Soils Surrounding Typical Industrial Areas of South Shanxi Province, China
Source: Toxics. 2025 Nov 16;13(11):984. doi: 10.3390/toxics13110984 (PMC12656453; doi:10.3390/toxics13110984)
Supplement: Supplementary file 1 [file toxics-13-00984-s001.zip › toxics-3957889-supplementary.pdf]

Supplementary Materials

for

Distribution, Sources, and Risks of Heavy Metal Contamination in Farmland Soils Surrounding Typical Industrial Areas of South Shanxi Province, China

Ying Zhao [1], Yirong Ren [1] and Fei Wang [2,*]

[1] Department of Chemistry and Chemical Engineering, Jinzhong University, Jinzhong 030619, China; shadowying210@163.com (Y.Z.); renyirong621@163.com (Y.R.)

[2] School of Life Science, Shanxi University, Taiyuan 030006, China

* Correspondence: nemo@sxu.edu.cn

Table S1. The geochemical background value for heavy metals [26].

	Pb	Cd	As	Hg	Cr	Zn	Cu	Ni
Background value (mg/kg)	14.7	0.102	9.1	0.023	55.3	63.5	22.9	29.9

Table S2. The classification standards for soil heavy metal pollution [29-30; 32-33].

Index	Category	Description
Geo-accumulation index (I_{geo})	$I_{geo} \leq 0$	Essentially uncontaminated
	$0 < I_{geo} \leq 1$	Slightly contaminated
	$1 < I_{geo} \leq 2$	Moderately contaminated
	$2 < I_{geo} \leq 3$	Moderately to heavily contaminated
	$3 < I_{geo} \leq 4$	Heavily contaminated
	$4 < I_{geo} \leq 5$	Heavily to extremely contaminated
	$5 < I_{geo}$	Extremely contaminated
Single factor index (P_i)	$P_i \leq 1$	Essentially uncontaminated
	$1 < P_i \leq 2$	Uncontaminated to slightly contaminated
	$2 < P_i \leq 3$	Slightly contaminated
	$3 < P_i \leq 5$	Moderately contaminated
	$P_i > 5$	Heavily contaminated
Nemerow comprehensive index (P_N)	$P_N < 0.7$	Safe domain
	$0.7 \leq P_N < 1.0$	Precautionary domain
	$1.0 \leq P_N < 2.0$	Slightly polluted domain
	$2.0 \leq P_N < 3.0$	Moderately polluted domain
	$P_N > 3.0$	Heavily polluted domain
Ecological risk index (RI)	$RI \leq 110$; $E_r^i \leq 40$	Low risk
	$110 < RI \leq 220$; $40 < E_r^i \leq 80$	Moderate risk
	$220 < RI \leq 440$; $80 < E_r^i \leq 160$	Considerable risk
	$440 < RI \leq 880$; $160 < E_r^i \leq 320$	High risk
	$880 < RI$; $320 < E_r^i$	Extreme risk

Table S3. Parameters and descriptions for the health risk assessment [36-37].

Parameters	Description	Units	Values
$IngR_s$	Ingestion rate of soils of children (adults)	mg/day	200 (100)
ED	Exposure duration of children (adults)	year	6 (24)
EF	Exposure frequency of children (adults)	day/year	250
BW	Average body weight of children (adults)	kg	15 (70)
ABS	Dermal absorption factor	unitless	0.13
AT	Average exposure time for carcinogenic (non-carcinogenic) effect	day	25550 (365×ED)
$In\mathit{h}R_s$	Inhalation rate of soil of children (adults)	m^3/day	8 (20)
SA	Exposed skin area of children (adults)	cm^2	2800 (5700)
AF	Adherence factor for children (adults)	mg/cm^2/day	0.2 (0.07)
PEF	Particle emission factor	m^3/kg	1.36E+09

Table S4. Reference dose (RfD) and slope factor (SF) of heavy metals from different pathways [36-37].

	RfD (mg/kg/d）			SF (mg/kg/d）		
	Ingestion	Dermal absorption	Inhalation	Ingestion	Dermal absorption	Inhalation
Ni	2.00E-02	5.40E-03	2.06E-02	-	-	-
Cu	4.00E-02	1.20E-02	4.02E-02	-	-	-
Zn	3.00E-01	6.00E-02	3.00E-01	-	-	-
Hg	3.00E-04	2.10E-05	8.57E-05	-	-	-
Cd	1.00E-03	1.00E-05	1.00E-05	5.10E-01	2.00E+01	6.30E+00
As	3.00E-04	1.23E-04	3.00E-04	1.50E+00	3.66E+00	1.51E+01
Cr	3.00E-03	2.50E-04	2.86E-05	5.00E-01	-	4.20E+01
Pb	3.50E-03	5.25E-04	3.50E-03	-	-	-
